# Food socialization of children with Prader-Willi syndrome: an interdisciplinary problematization

**DOI:** 10.3389/fnut.2023.1177348

**Published:** 2023-06-06

**Authors:** Amandine Rochedy, Marion Valette, Maithé Tauber, Jean Pierre Poulain

**Affiliations:** ^1^Université Toulouse—Jean Jaurès, Toulouse, France; ^2^UMR5044 Centre d'Etude et de Recherche Travail, Organisation, Pouvoir (CERTOP), Toulouse, Midi-Pyrénées, France; ^3^Reference Center of Prader-Willi Syndrome and Other Syndromes with Eating Disorders PRADORT, Children’s Hospital, Toulouse, France; ^4^UMR1295, Centre for Epidemiology and Research in Population Health (CERPOP), Toulouse, France; ^5^INSERM UMR1291 Institut Toulousain des Maladies Infectieuses et Inflammatoires, Toulouse, France; ^6^Chair of “Food Studies: Food, Cultures and Health”, Taylor’s Toulouse University Center, Taylor’s University, Kuala Lumpur, Malaysia; ^7^Faculty of Social Sciences and Leisure Management and Centre for Asian Modernisation Studies, Taylor’s University, Kuala Lumpur, Malaysia

**Keywords:** food practices, food socialization, interdisciplinarity, neophobia, Prader-Willi syndrome, children, autism

## Abstract

Eating “disorders” of people with Prader-Willi syndrome are frequently reported in the biomedical literature. The eating behaviors are presented as a syndrome-specific trajectory over the course of a lifetime. Infants initially show anorexic behavior, which then develops into hyperphagia that lasts from childhood to adulthood and is characterized by strong cravings for food and relentless thinking about it. However, the sociocultural determinants of these food practices are not fully understood. In the first section of this article, we carry out a literature review of medical articles published on disordered eating in children with PWS. The second section draws on a social science perspective and offers an interdisciplinary problematization using the concept of food socialization. To conclude, the third section explores the challenges facing research and new questions that emerge from the alternative problematization that is the PWS Food Social Norms Internalization (FSNI) theory.

## Introduction

Prader-Willi syndrome (PWS) is a rare disease characterized by disordered eating. Manifesting as difficulty sucking and swallowing breast milk or formula in newborn infants, the disease primarily shows up as hyperphagia as the subject gets older, often causing severe obesity. Medical treatment focuses on the difficulties encountered by affected individuals in controlling their eating behavior and the consequences this has on family life. Such an approach emphasizes that, although sociable, children with PWS tend to experience issues surrounding socialization in large part due to their pervasive thoughts (1) about food and their compulsive eating (2, 3).

These issues pose a challenge for the sociological study of their food habits viewed through two aspects of socialization. The first of these relates to social interactions that take place during mealtimes. The second refers to the internalization of social norms that teach children to eat appropriately in a range of social settings. Thus, food socialization is central to a child’s psychosocial development and in the development of their identity (4, 5).

The socialization issues experienced by this population can be examined based on: (i) how a child’s food likes and dislikes are constructed in relation to the social groups to which the child belongs and, more generally, the uses of food in wider society; (ii) the internalization of social norms, teaching children to behave “properly” at the table: rules of precedence, cleanliness, quantity ingested, etc.; and (iii) the process of building a social identity.

In children with PWS, we focus on the learning obstacles encountered during the socialization process. One of the frameworks for understanding food socialization is based on the theory of neophobia (6–11), which describes the cycle through which children experience a phase of narrowed food diversity following the construction of their own food repertoire. Recent studies on the socialization of children with autism spectrum disorders (ASD) have shown deviations in the typical process (11–13). Children are assumed to be emerging from the cycle of neophobia when they gain an awareness of other people and that their refusal to follow certain rules may lead to negative consequences.

Through an articulation of the PWS reading grid of nutrition with that of food learning developed by food sociologists, this article proposes to describe, identify and understand the (dys)functions within food socialization in these children whereby they develop the ability to improve their autonomy and enhance the quality of life for both them and their families.

This article is a “Conceptual Analysis” which aims to describe and to problematise in an interdisciplinary perspective the question of the eating disorders of the PWS children by focusing on the internalization of the social norms (5, 14–16). The internalization of food behavior norms allows a child to eat in society. Theses norms concern table manners, such as the use of tools, body control, postures, identification of common and individual spaces at table as well as chewing habits, control of body noises and the place given to pleasure. Learning of these rules takes place during social interactions both during and beyond mealtimes at home with immediate family members (i.e., parents, siblings, domestic helper). Learning also takes place outside of the home, with extended family members, friends, or even at school canteens which drives to internalization of these norms.

In the first section of this article, we carry out a literature review of medical articles published on disordered eating in children with PWS. The second section draws on a social science perspective and offers an interdisciplinary problematization using the concept of food socialization. To conclude, the third section explores the challenges facing research and new questions that emerge from the alternative problematization that is the PWS Food Social Norms Internalization (FSNI) theory.

## The peculiar trajectory of food practices in children with PWS

PWS is a rare genetic neurodevelopmental disorder affecting around one in 20,000 newborn infants (17, 18). Eating disorders are a dynamic characteristic of the syndrome, with affected infants displaying anorexic behaviors, followed by the development of hyperphagic behaviors driven by strong cravings for and persistent thinking about food. Very early in childhood, this preoccupation with food pervades daily life to such an extent that some researchers have proposed looking at it as a “food addiction” (3, 19). There is currently no medical treatment available for this condition, and early multidisciplinary management is based on food control: first by optimizing calorie intake from birth, followed by stringent control over access to food in order to prevent severe obesity. Strategies for applying this control are offered and supported by healthcare professionals and implemented by the subjects’ families on a day-to-day basis.

The work of the Florida group (20) identifies seven nutritional phases. *In utero* (phase 0), fetal movements are reduced and there is an excess of amniotic fluid (polyhydramnios) probably due to a lack of swallowing. At birth (phase 1a), severe hypotonia is observed, with a certain weakness in sucking, a lack of appetite and difficulty in gaining weight (failure to thrive), prompting nasogastric tube feeding in 80% of cases (18, 21–23). Between 9 months and about 2 years (phase 1b), feeding becomes easier and the child grows at a normal rate. Then, despite no increase in calorie intake, the child begins to gain excessive weight (phase 2a), followed by increased demand and searching for food (phase 2b). In the absence of control, severe obesity rapidly occurs due to excessive food intake, along with pervasive thoughts about food, active searching for food, overeating at meals and lack of satiety (phase 3). In adults (phase 4), some individuals are able to feel full. The overeating and lack of satiety in phases 3 and 4 have been studied extensively (24, 25). The overwhelming preoccupation with food and the impulsive urge to eat are manifested both at mealtimes and at other points during the day, leading to major behavioral issues such as temper tantrums, hitting out at others, repetitive behaviors, etc. (1).

Food therefore plays a central role in PWS, profoundly disrupting daily life. Families find themselves obliged to restrict access to food for children with PWS on an almost permanent basis, both in and outside the home. However, the seven nutritional phases of the eating trajectory are commonly described without consideration for the social environment of the person with PWS.

Three main paradigms are used to elucidate the food practices of people with PWS: genetic, endocrine, behavioral. They coexist without contradicting one another in any way and all take a developmental approach.

### Genetic paradigm

Based on its original 1956 description (26), in 1989 PWS was the first condition to be identified as being caused by parental genomic imprinting (27–29). The clinical phenotype is due to a loss of expression of certain genes of paternal origin in the chromosomal region 15q11-q13. In infants diagnosed with PWS, this absence of expression results from a paternal deletion (around 50%), a maternal disomy (around 50%), or much more rarely a deficit of genomic imprinting or a translocation (less than 3%) (18). It is now well established that the mutation of a single gene on the paternal chromosome, *SNORD116*, reproduces the phenotype of PWS in humans and mice, with a developmental anorexia-hyperphagia trajectory (30–32). The expression of this gene is variable during development, while generally having a tightly regulated hypothalamic expression. In mice, the full developmental trajectory of anorexia-hyperphagia is achieved when *SNORD116* expression in the hypothalamus is blocked after weaning.

The mutation of another gene, *MAGEL 2*, reproduces the first phase of PWS in mice and humans, with neonatal hypotonia and anorexia. People with an isolated *MAGEL2* mutation have Schaaf-Yang syndrome, which is considered to be related to PWS, and are frequently diagnosed as having ASD.

### Endocrine paradigm

The complete PWS phenotype can be explained by an impaired hypothalamic development and dysfunction. The *SNORD116* and *MAGEL2* genes are involved in the establishment, function and ontogeny of hypothalamic neurons, which secrete oxytocin (OT) (33–36). In addition, studies on neurons derived from induced pluripotent stem cells (iPSc) obtained from patient samples have demonstrated a failure to regulate the maturation of several hypothalamic hormones and other hormones such as insulin and ghrelin. This maturation defect is linked to a deficiency in the proconvertase 1/3 enzyme (PC1/3) and at least partially explains the endocrine abnormalities of PWS, particularly obesity with hyperphagia due to a defect in the maturation of proopiomelanocortin (POMC), other pituitary hormone deficits, and problems with insulin, OT, and ghrelin maturation (37). Eating disorders may be linked to abnormalities in OT and ghrelin: these hormones interact very closely and are strongly involved in brain development and the regulatory mechanisms of the dopaminergic system involved in the reward system. OT is secreted by hypothalamic neurons in the paraventricular and supraoptic nuclei, while ghrelin exerts most of its metabolic and appetite-regulating effects in the hypothalamus. The two hormones also have peripheral effects, particularly on the digestive, cardiac and bone systems, among others. In people with PWS, plasma total ghrelin levels are elevated from birth and remain high throughout their lives (38). Ghrelin comes in two forms: acylated (AG; binding of a fatty acid to the peptide chain) and un-acylated (UAG). AG has a strong orexigenic effect (leading to it being known as the “hunger hormone”), whereas UAG inhibits the effects of AG and therefore has an anorexigenic effect. The ontogeny of the ghrelin system is different in PWS and may explain the anorexia-hyperphagia trajectory and the metabolic disorders (39). From our perspective, hyperghrelinemia could also be involved in obsessive thinking about food and compulsive eating described in people with PWS. Indeed, ghrelin is involved in addictive behavior, kleptomania, and certain neurodegenerative pathologies. In mice, it has been shown that ghrelin peaks are regulated by the frequency of meals. The ghrelin system is involved in the “food entrainable oscillator”—a system that is modulated by food intake and induces a motor response that makes it possible to seek and obtain food. People with PWS have an “ultra-sensitive food clock,” possibly related to high ghrelin levels, driving very precise anticipation of mealtimes. OT is involved in both the control of satiety and behavioral disorders (social skills, regulation of emotions). The abnormalities of these two very intricately connected hormonal systems in subjects suffering from PWS go some way to explaining their eating behaviors. OT plays a fundamental role in establishing the sucking reflex and the pathways involved in orality, as well as in the regulation of the neuro-vegetative system (40). These functions are deficient in PWS to varying degrees. Ghrelin and OT work through receptors in the brain, particularly the dopamine neurons involved in the reward system, and in many other organs (41).

### Behavioral and eating disorders

Oral disturbance is present in PWS from birth and persists throughout the sufferer’s lifetime. Regulation of the sucking-swallowing reflex is present from birth, and voluntary control subsequently occurs with learning. The maintenance and development of sucking skills help to establish the neural networks involved in the control of feeding and is also utilized in social learning. The orbitofrontal cortex contains neurons that respond to food stimuli and those that respond to social stimuli, with interactions between these two neuronal populations (42).

From the age of 7–8 years, food impulsivity echoes general impulsivity. In adults, compulsive eating can be present alongside other addictive behaviors, mainly smoking and, more rarely, alcohol abuse. Alongside the theft and storage of food, compulsive theft similar to kleptomania and the storing/collection of objects is often observed. Notably, these behaviors described in relation to food (frustration, bargaining, rigid thinking, difficulty in diverting attention, perseverance, theft, anger, etc.) have been observed to be intricately intertwined with all other areas of daily life. A hyperphagia questionnaire specific to PWS was developed (24, 25), dealing with: (i) behavior: describing the times, frequency, actions and bargaining involved in obtaining food; (ii) drive: describing the strong need or desire (impulsivity) to speak about or consume food, as well as the difficulties encountered in diverting the sufferer’s attention from food, their perseverance, and avoiding frustration and anger; and (iii) severity: describing the extent to which the person is invaded on a daily basis by thoughts, words and actions relating to the search for or consumption of food. A high level of anxiety and restlessness before sitting down at the table has been observed, with individuals suffering from PWS becoming preoccupied about mealtimes and the quantity and quality of their food. Meal intake is either very rapid and even voracious, with significant risks of aspiration and suffocation or, conversely, excessively slow. People with PWS have been known to carefully collect every last crumb, entirely clean their plates, repeatedly scrub at the bottom yogurt containers and make exaggerated comparisons with the contents of the plates of other people sitting around the table.

Despite a proven deficit in executive (e.g., problem solving) and planning functions, people with PWS can develop highly elaborate strategies to carry out the steps necessary to access food that has been locked away. Parents often note that their child’s intelligence is entirely directed toward finding and consuming food, although it is possible to divert their thoughts with other pleasant activities (games, television, etc.).

### Family management of feeding practices

The announcement that a child is suffering from a serious pathological condition can significantly disrupt family life. The day-to-day strategies put into place to cope with disabilities or the child’s differences have been studied within families (12, 43, 44), especially among mothers (45). With PWS, families and caregivers regularly discuss the adjustments that need to be made to avoid either imposing excessive restrictions on food, or relaxing these restrictions “too much” or “too frequently.” According to the literature, the earliest family strategies thought to be “most effective” for food and weight management were: locking up food, constantly monitoring the child while he or she eats, and offering only low-calorie foods as snacks (46). More recent strategies have been suggested, including maintaining the child’s involvement in activities and the use of routines within the family unit (47). An American team proposed an adage concerning meals: “No doubt, no hopes, no disappointment”^1^ to reassure and reduce the anxiety of people with PWS, especially those living in an institution. An environment of benevolent empathy has been shown to help contain, reassure and support such people. Conversely, a lack of a supportive framework or displays of excessive authority significantly worsen their behavior and anxiety levels. The important thing appears to be supporting them in their efforts to acquire autonomy in areas of their lives, but always with the understanding that the objective is not full autonomy. For example, talking about and anticipating any temptations or opportunities that may arise (in stores, vending machines, etc.) helps people to achieve a feeling of independence and a legitimate degree of freedom. In addition, the healthcare professionals at the PWS reference center^2^ remain attentive to vulnerable families with poor adaptive skills (48).

## Food socialization: a new point of entry for understanding eating practices

Eating behavior is a complex phenomenon. It results from the interactions between more or less “genetically” controlled or biologically overdetermined predispositions and social learning, which itself varies from one culture to another and can even be affected by a person’s social position. From birth, the role of influence is powerful. The malleability of behavioral patterns allows for adaptation to a range of contexts. Moreover, learning affects predispositions, either amplifying or reducing their plasticity. The ability to implement eating behaviors suitable for a given social context and culture therefore results from the interaction between dispositions and learning. “Food socialization” refers to these interactions that occur over the course of a child’s development and lead to the capacity to adopt behaviors adapted to social contexts.

### The place of culture in eating habits

Human eating behavior is determined by biological and sociocultural factors. It is primarily determined by the biological status of the species as an omnivore. This is characterized by possibilities, digestive capacities, and constraints (the methods of breaking down food into nutrients, the synthesis and storage of certain nutrients, etc.). Certain reflexes should also be noted: sucking, swallowing and even preferences and dislikes for certain flavors. But eating behavior is also influenced by sociocultural processes. Although omnivore status defines predispositions and incapabilities, it has certain areas of freedom within which choices can be made without biological consequences (at least in the short term). Food can be ingrained in social mores. Within the broad set of potential foods, each culture selects specific foodstuffs and develops an “edible order” (4). How meals are presented and eaten, table manners, and rules of precedence are all ways a society presents its values at a given point in time (50–53). Meals provide opportunities for children to internalize the social norms and rules of conduct that prepare them to behave “properly.” These “social food spaces” have areas of freedom, giving rise to the expression of cultural diversity where the processes of social differentiation take place (5).

Yet although the distinction between biological and sociocultural determinants is useful, it is also appropriate to examine the interactions between them. Culture has an impact on genetics by participating in modes of selection, transmission and dissemination of genes in society. Examples of this are kinship rules (54). Their specific details (prescriptions and prohibitions) affect the availability of certain characteristics at the level of population genetics and in the processes of phenotype expression or non-expression. Indeed, a new body of knowledge has arisen to examine these very issues: nutrigenetics (55–57). This field is restructuring the relationships between the biological sciences and the human and social sciences (58, 59). Interestingly, *in utero* conditioning has been highlighted, with studies finding that the taste of amniotic fluid changes with what the mother eats to the extent that the child becomes accustomed to frequent flavors in the food culture into which he or she will be born (60). The culture in which an individual is immersed therefore intervenes even before birth.

### Food at the heart of the socialization process

Genetic heritage and the food model of the society into which a person is born are therefore somewhat established without any choice being made by the individual. Babies come into the world in a state of “dependence” and “incompleteness” (61). Initial programming enables them to suckle, digest breast milk and prefer sweet flavors, but everything else has to be learned, from using the senses to eating behaviors through to the acquisition of table manners. This learning takes place in sociocultural contexts and through social interactions. It is necessary to ensure the processing of information and especially to semanticize it, or to give it meaning (62). Learning about food also make it possible to attribute a scale of magnitude to sensations, which are both personal and derived from the norms of a person’s social group. Food lies at the heart of the socialization system. When learning to eat, children set up behavioral modes useful for implementing and controlling this biological activity and simultaneously internalize the value system of the social group into which they were born.

Research in psychology has elucidated the modes of passing from sensation to perception, as this is essential for generalizing, categorizing, and ultimately constructing the “lived world.” This transition is a decisive step in “learning to eat” as it allows an individual to build a repertoire of edible products and appropriate behaviors. This type of learning occurs through observation and imitation of adults and peers. The appropriation of food repertoires takes place over the course of the different socialization stages and in a range of social contexts (family, school, recreation center, etc.). Sociologists and psychosociologists emphasize that food learning begins early on in life within a web of emotional and relational contexts. Emotion and hedonism thus play a major role for children and remain the driving force behind their relationship with food over the course of their lifetime. For this reason, cognitive factors, though undeniably important, are not enough to learn or modify eating practices. Here, practices that are learned take precedence over those that are innate.

From the earliest days of sociology, food and food socialization have always occupied a special place. Émile Durkheim (63) sought to mark out the territory of this new discipline and distinguish it from biology and psychology. He proposed two methods for defining its focus on “social facts.” The first definition specified what could not be deemed a “social fact.” Food was the first example given because it was “too biological.” The second definition listed the conditions for inclusion within sociology and among them was food once again, this time because of the rules of conduct that “are imposed on the individual from the outside.” Food was therefore excluded from sociological study when it came too close to biology but included when it came to the customs, rules and social norms that defined its implementation in society. Durkheim’s objective was to delimit an autonomous epistemological space in which he sought out the root of one social fact in another. Food is central to socialization and the transmission of these systems of norms from one generation to the next. It is thus part of a process of social integration and regulation that enables an individual to find his or her place in a social group and to be recognized as a member of the group. Sociology has therefore often focused on the social “institutions” that frame meals (16, 64, 65) and the social functions for which eating behaviors provide support.

Another tradition emerged from the work of Marcel Mauss (66) and his concept of the “techniques of the body” viewed in their social, psychological and biological dimensions. The conditions for an interdisciplinary approach came together in this pioneering work, but the focus remained on the influence of the social over the biological, with psychology relegated to a secondary role of articulation. These two traditions still weigh heavily on sociology today.

The traditional concept of the social fact has continued through Bourdieu’s theory, with concepts of “incorporation” and “habitus” for identifying how social position influences bodies and tastes (67, 68). The concept of habitus juxtaposes two sides, one passive and the other active. The first refers to internalization during the socialization phase and the other ensures the organization and cognitive structuring of a situation after the end of this phase (69). From this perspective, the notion of “disposition” refers to a propensity to act. The emphasis, however, is more on the social origins of individuals and the consequences for their choices and tastes than on the construction of dispositions.

The second tradition is clearly interdisciplinary, and this is exemplified by the research on neophobia. A new definition of the concept of “incorporation” bases itself on what a person might imagine is happening during the act of ingesting food, thereby taking into account its psycho-sociological consequences. Incorporation from this perspective has two dimensions: (i) imaginary: symbolic appropriation, signs, norms, etc., and (ii) social: sharing norms and representations (7, 70).

However, both traditions are primarily concerned with the results of socialization and less with the dynamics of the process itself. It was not until the development of the sociology of childhood (71) and then of its connection with food (72–77) that the focus began to shift to the processes of socialization.

Psychology, however, began to examine childhood development very early on. Three major theories, all of which complement one another and which form one part of the whole, have dominated the landscape (78): a psychoanalytic approach focused on affective and sexual development (79–81), a cognitive theory of psychology (82) and a psychosociological perspective (83). These three theories share a conception of development as occurring in stages. This refers to a succession of stages during which a person’s internal organization and functioning are more or less stabilized. The stages follow on from one another, each incorporating the features of the previous stage in an ever larger and more complex structure. Looking at these three theoretical perspectives from the outside reveals their dual complementarity. Firstly, when one theoretical framework becomes partially effaced, another increases in importance. One example of this is the lessening in importance of the psychoanalytic “latency period,” which results in a reduction in the libidinal problematic and an increase in the importance of intellectual and cognitive development. Secondly, each theory produces information about the same stage that sheds light on aspects that complement each other (84). Erik Erikson (85) can be credited with the idea that social identity comes as a result of a developmental process through various successive stages that extends over a person’s entire life.

### Food neophobia, a tool for studying socialization

The concept of “food neophobia” has been used in two distinct analytical scales. From an anthropological perspective, it accounts for the ambivalence in the relationships that eaters maintain with food (fear of the unknown versus the search for novelty). Human eaters are faced with what has been called the “omnivore’s paradox.” The inability to synthesize certain nutrients requires them to have a varied diet, yet these foods may be potentially dangerous. Eating can therefore fluctuate between a fear of the unknown, prudence, the anxiety of incorporation (neophobia) and the search for novelty (neophilia). Human eating behaviors thus often manifest themselves in a back-and-forth between neophobia and neophilia, thereby generating a certain level of anxiety. Linked to incorporation, this is regulated by our culture surrounding food, which establishes a system of what is edible and associates symbolic representations with food (5).

**Figure 1:**
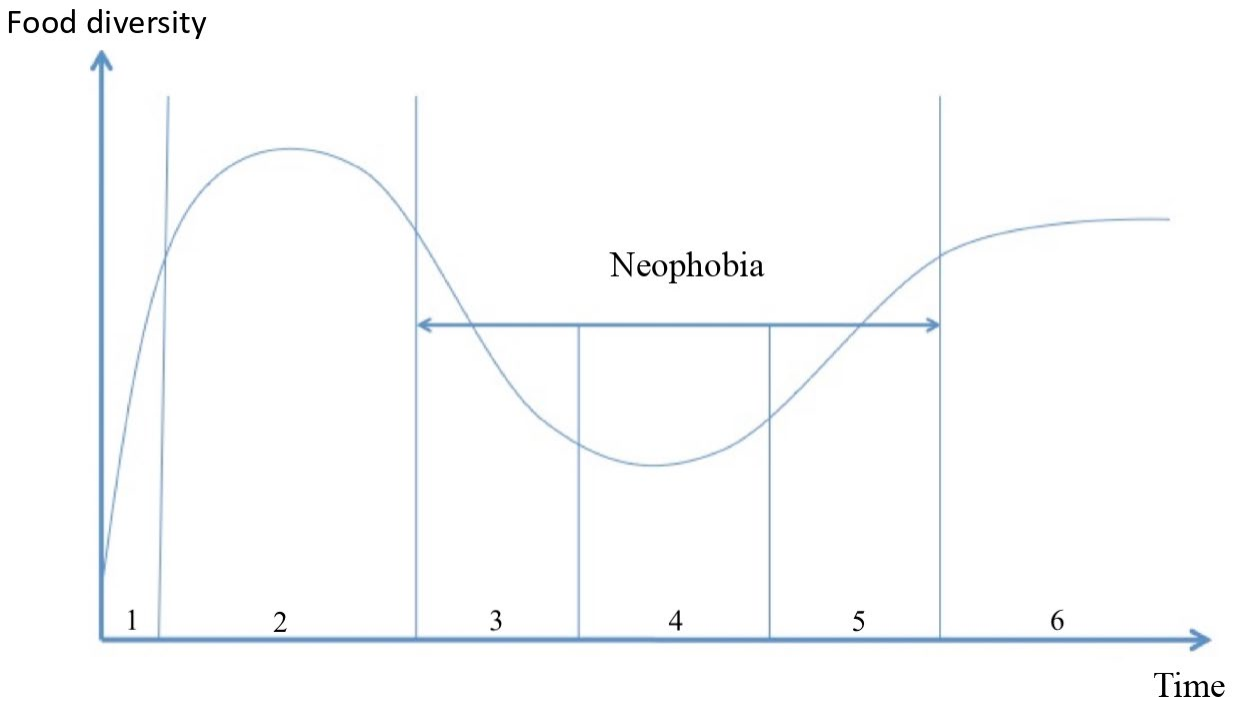
From milk to the adult food repertoire.

The process of neophobia can be analyzed from two perspectives: the inter-individual variations in the development of a food repertoire and the intensity of neophobia. In the absence of a quantitative survey, our current knowledge about typical children offers an ideal-typical view (6). The process of neophobia can be broken down into several stages (Figure 1)(9): (1) A milk diet, (2) food diversification followed by the neophobia itself characterized by (3) closing off of the food repertoire, (4) stabilization, (5) reopening of the food repertoire and, finally, (6) the establishment of a more or less permanent food repertoire. Breaking down the neophobia process into stages makes it possible to identify pivotal moments and gain a deeper insight and therefore to act in more helpful ways.

Neophobic behavior in children is observed from the age of 2 years, albeit with individual variations (7). More than three quarters of children go through this stage of neophobia in a more or less pronounced way, classified into four degrees of severity (8).

Research in children with ASD showed that neophobia revealed a number of deviations from typical processes (13). In the first sub-population, children presented with “typical” neophobia, but the intensity varied in strength. In the second subset of population, neophobia did not present in its classical way. In the “progressive” form, neophobia gradually increased from birth, while in its “late” form, neophobia started occurring at the age of 7, and in its “neophile” form, children did not exhibit neophobic behavior. This typology, in terms of the construction of the food repertoire, indicates that phenomena that have often been identified as specific to the autistic population in fact stem from deviations—prolonged, intensified, a combination of both, absence or delay—in the construction of food neophobia and its processes. It is therefore important to refer to food neophobias in the plural. The study highlighted a number of social and medical factors in the types of processes (12). ASD is associated with the genetic abnormalities of PWS (86) and similar behavioral symptoms are observed in the two populations (87). To cite just one example: altered social interactions with a specific weakness in interpreting social information and in responding to them. Therefore, it will be interesting to better understand the place of neophobia in the development of eating practices among children and adolescents with PWS.

### Food neophobia and stages of development

Poulain (9) synthesized a number of theories surrounding the development of children with neophobia. To describe the dynamics of establishing a food repertoire, we can distinguish different stages, although they remain closely related to their preceding stages.

Initially, children consume only one type of food: milk. According to psychoanalytic theory, this is the first part of the oral stage, known as “gratifying” swallowing, during which the child and mother maintain a symbiotic relationship.

The second phase, the “transition” between milk and solid food is characterized by the food repertoire starting to open up. First, new foods are introduced in liquid or semi-liquid form (purée) alongside milk. Solid foods are then introduced. In this stage, many children will eat almost anything offered to them and show few food preferences. The food repertoire is wide open, only limited by the child’s culture and family. This period is the second part of the oral stage, which begins with teething and during which the child experiences the pleasures of biting and chewing. During the two parts of the oral stage, the child puts many objects in its mouth and taste is marked by low discrimination, with an appetite for sweet flavors and a more or less clear rejection of sour and bitter tastes.

The onset of neophobia may occur during teething. These children will then experience the sucking/biting dilemma. They feel the urge to bite the mother’s nipple, but if they do, she withdraws her breast. They thus enter a world of ambivalence about: (i) a desire that forms the basis for the “splitting of objects” and access to the “object world,” and (ii) the objects which, from then on, can be “good” and “bad” at the same time (88). As opposed to the previous stage, neophobia is characterized by a narrowing of the edible space. At this stage, children refuse new foods and often temporarily abandon foods that were once deemed acceptable (11). In doing so, each child asserts personal tastes and preferences and a decision whether or not to eat (89). The intensity of the reduced number of foods and the duration of the phase varies from individual to individual. The neophobia cycle has three stages: narrowing, low stabilization and a reopening of the edible space. During these stages, social interactions take place with the adults who provide food and fellow eaters. They contribute to the socialization and construction of the personal identity of children through the internalization of a set of norms and rules relating to eating and their eating behavior itself. The appropriation of these norms contributes to the recognition of an individual as a member of the social group, the family first of all and then the other social groups to which he or she belongs. Children thus become recognized and accepted as members of the groups in which they socialize. Moreover, some leeway is acceptable in applying the group’s norms and rules, and this allows children to develop a style that contributes to the process of constructing a personality. Developmental theories have identified phases of opposition to parental authority during which children assert themselves as individuals. Neophobia arises at the time of one of these phases and is therefore seen as an expression of opposition that is a normal stage of development. For Henri Wallon, for example, the period of 3 to 6 years old is the “stage of personalism,” during which children tend to oppose adults in a kind of “negativist crisis.” At this stage, the child is expressing ambivalence about a model embodied by adults. Wallon distinguished three sub-stages to this stage. In the first, children clearly oppose the adult. In the second, their behavior becomes much more accommodating, and in the third and final stage, they try to imitate the adult (83). Some have termed this period the “no phase” (90). The result of a long process of somato-psychic maturation, it opens the way to human communication and initiates the process of developing the personality (91). In this context, food plays a particularly important role. By refusing certain foods, children engage in power struggles with their parents and seek recognition as individuals with likes, dislikes and preferences. This recognition also involves the internalization of behavioral norms that frame the act of eating. By affirming personal choices, they begin constructing their food repertoire. Chiva (62) assumed that neophobia contributed to the process of attributing meaning to sensory experience and precipitated the passage from foods viewed as being “for us,” referring to the family, toward the foods that are “for me.” To semanticize means to attribute meaning and valence to a sensory experience on hedonic and moral scales. It is essential to the individual’s orientation and development of future choices, and it complicates and gives meaning to the rudimentary basic flavors of sensory psychology.

After the reopening stage, children assert what will essentially be their permanent food repertoire. To do this, they select from the range of foods available within their culture and family. The origins of food preferences and dislikes have not been settled and may also be determined by social influences, psychological issues, allergies, deficits in digestive ability, and so on. Advances in epigenetics could help clarify this issue.

A systematic approach to the successive stages of the three main theories of child development with regard to the food neophobia cycle is put into perspective with the food peculiarities of children with PWS (Table 1).

**Table 1:** Neophobia processes, developmental stages in child psychology, and food practices of children with PWS.

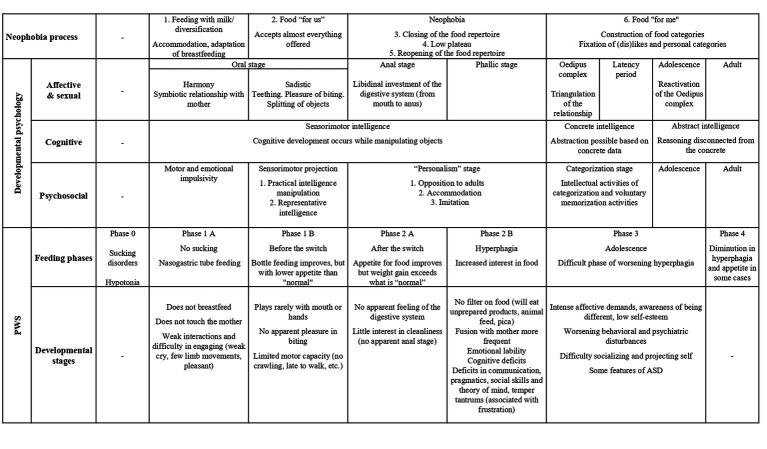

This systematic approach to the theories and their dimensions has been fundamental in the development of our research questions and the problematization of PWS.

## Toward a PWS food social norms internalization theory

This Food Social Norms Internalization (FSNI) theory articulates three main concepts: the internalization of food norms, neophobia and familialization. It claims that the incorporation of social food norms that takes place as part of social interactions with family members (parents, grandparents, siblings, etc.), is disrupted by PWS. This disruption is primarily derived from the bio-psycho-developmental side of the syndrome itself, mainly the trajectory of eating disorders, from anorexia to hyperphagia. Secondly, it comes from the structure of control systems set up by caregivers to manage the issues facing affected individuals in controlling their eating behavior.

Food Social Norms are rules related to table manners such as the use of tools (spoons, forks, knives, chopsticks, etc.), the identification of shared and personal spaces at the table, postures, chewing habits, body control, body noises, as well as the importance attached by families and cultures to pleasure. The appropriation of these social norms and rules allows a child to eat in society “normally.” “Neophobia” refers to a developmental phase in which children may experience food restrictions, refusing certain foods and coming into conflict with their food caregivers. This “neophobia” phase plays an important role in the internalization of social norms and more generally in the socialization process. The concept of “familialization” was first used to analyze the transfer of activities and responsibilities from the family to state or private care systems. “De-familialization” describes the transfer of activities from the family to care systems and “familialization” from care systems to the family (92). This concept was used to develop international comparisons (93–95) and to study the consequences for gender equality (96). But “familialization” has also an alternative meaning. In a situation where one family member faces a chronic illness, their family members will typically use information and advice provided by health professional experts to re-structure their everyday life, including the distribution of parental roles (97, 98). This is the meaning to which we refer within FSNI theory. The familialization process therefore relates to how family members take on messages and advice formulated by health actors at the time of diagnosis and during the management of patient care.

**Figure 2:**
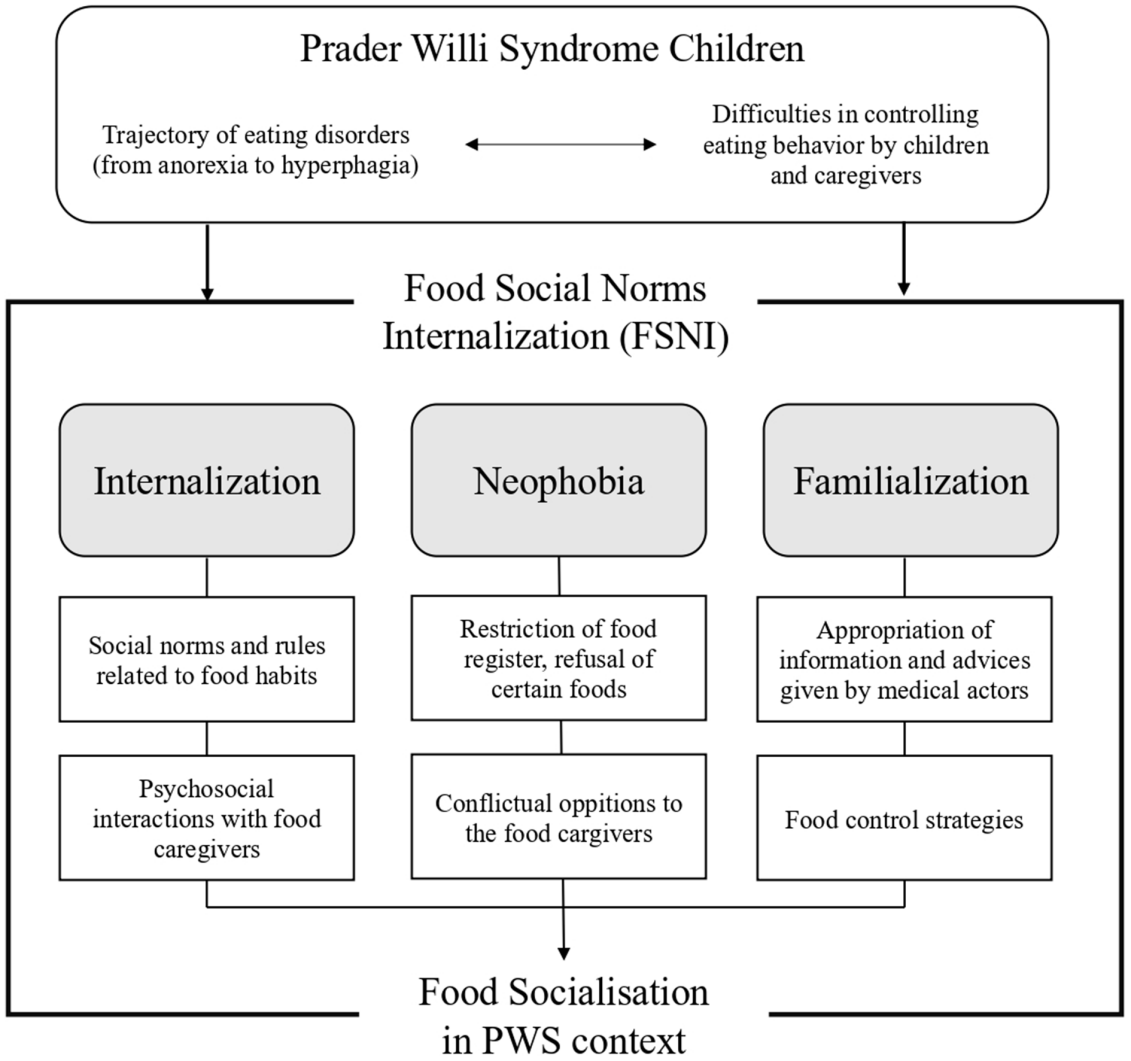
From eating disorder to food socialization. Internalization of food social norms in the context of Prader-Willi Syndrome.

For PWS children, the process of food socialization is much more complex than for their unaffected peers. The syndrome places eating behavior at the absolute center of family life. The syndrome itself modifies appetite and eating behavior, and disturbs the neophobia process. It also dictates the different ways to enact control over eating behavior as implemented by family interactions (familialization). This is the highly specific context of food socialization among PWS children (Figure 2). How do family members provide meaning to social norms surrounding food and reformulate them? How does this information then translate into a food management strategy? Who are the players and what are the roles played within the family to manage any food strategy? All these questions are linked to the role of familialization in shaping food socialization among PWS children. Exploring these concerns may also provide an insight into the extent that strategies put in place by parents could either facilitate or disrupt learning, the internalization of norms and food empowerment among PWS individuals. This theoretical framework enables the development of new research focusing on the process of the internalization of social norms and their specific nature among PWS children. What creates an obstacle to learning in children’s behavior and to their interaction with their surroundings.

This re-problematization opens up new avenues of research likely to enable the development of new ways of providing care. From research standpoint today, the four main active lines are:

- a genetic approach, identifying the specific genetic characteristics of people with PWS;
- the endocrine approach, which explores hormonal dysfunction due to abnormal hypothalamic development, which is said to more or less result from genetic configurations;
- a behavioral approach, describing the disorders and their changes running alongside psychosocial development; and finally,
- familial management of the care for PWS children.

By focusing on the issues posed by the internalization of social norms relating to food, the proposed re-problematization links the approach made to the eating disorder itself with the approach to family-based management. This new perspective emphasizes the role of social interactions in the process of internalization, including the conflictual ones.

Interdisciplinary questions will emerge from this view of the above classical approaches. For example, (i) the difficulties of internalization could be the starting point for a new description of the phenotype; or (ii) taking into account the stages of the PWS eating disorder trajectory, from anorexia to hyperphagia, developments could be made in adaptive and evolutive food education strategies. Last but not least is the traditional relationship between what is normal and what is pathological, which has shown how knowledge developed about a pathology can simultaneously further our knowledge on what is said to be normal (99, 100). As often, by studying pathology, we can expect a better understanding of normal processes. Examining eating disorders among PWS children as part of a general theory of socialization could be a way of advancing knowledge about the phenomenon of the internalization of food social norms among other eating disorders and even among typical children.

From the point of view of medical care and family management, the knowledge gained through such an examination could be used in at least two different ways. Firstly, on slowing down the pressure of external control by food caregivers and secondly on the “nutritionalization” of food experiences (the reduction of food to its weight and nutritional components) (101). Thus, food education and the daily food life of PWS children would leave more room for practices promoting the internalization of norms and the establishment of self-controlled routines. Conflicts between children and parents would not only be viewed as negative experiences, but also considered as a process of negotiation more likely to benefit the internalization of norms.

The perspective suggested would allow us to develop new methods of education and a comprehensive management for health professionals by integrating food social norms surrounding the concepts of internalization, neophobia and familialization. This would entail making efforts in training health professionals to include these concepts in routine care, by giving an insight into these concepts and taking into account all these dimensions for each child within a family context. This comprehensive approach may avoid or strongly mitigate any harmful effects of care and ensure the best possible level of food socialization.

Finally, some strategies already implemented successfully by some parents can be re-analyzed in the light of the FSNI theory and give rise to positive therapeutic education strategies involving the PWS community.

## Conclusion

Many scientific and practical issues converge on the food socialization of children with PWS. The neophobia approach restructures the issues on “eating disorders” and how to act because it focuses on issues surrounding the acquisition and learning of the systems of norms that allow a child to eat “normally.” By focusing on the difficulties of acquiring systems of norms allowing a child to eat “normally,” the neophobic approach reorganizes the problem of “eating behavior disorders.” This perspective opens up new ways of acting by inviting caregivers to move from external control to the search for conditions that could help the child to internalize norms and thus to empower, more or less depending on the case, his behavior. For example, this could be: patient family group discussion between parents on the question of neophobia and the function of “conflicts” in the food socialization process. Introduction into the clinical examination of questions relating to neophobia and food socialization by training health professionals who followed children and particularly young children and their families on the FSNI theory. This would help to facilitate and at least to prevent possible impaired neophobia.

This problematization invites to look at the interactions between biological determinants and determinants within the social environment. Hierarchies of determination may be revealed, opening up fresh insights and giving rise to new research questions on both sides. This will potentially create the conditions for an interdisciplinary dialog between professionals in food sociology, developmental psychology and pediatrics. The outcome of this interdisciplinary approach open news perspectives of research between medical sciences and social and human sciences. It may also help to redefine methods designed for intervention and care among PWS children and improve the support offered to their families.

## Data availability statement

The original contributions presented in the study are included in the article/Supplementary material, further inquiries can be directed to the corresponding author.

## Author contributions

AR, MV, MT, and JP contributed to formulating the scientific question, designing the research protocol, and drafting the manuscript. MT and MV inventoried the knowledge on eating disorders in children with Prader-Willi syndrome from a medical perspective. JP and AR did the same for food socialization from the perspective of the human and social sciences. All authors contributed to the article and approved the submitted version.

## Conflict of interest

The authors declare that the research was conducted in the absence of any commercial or financial relationships that could be construed as a potential conflict of interest.

## Publisher’s note

All claims expressed in this article are solely those of the authors and do not necessarily represent those of their affiliated organizations, or those of the publisher, the editors and the reviewers. Any product that may be evaluated in this article, or claim that may be made by its manufacturer, is not guaranteed or endorsed by the publisher.

## Footnotes

^1^ https://pittsburghpartnership.com/downloadable_educational_materia2.htm

^2^Since 2004, in France, PWS reference centre has three main goals: optimize patient’s diagnosis, care and management throughout life, inform and train health professionals and develop and/or support clinical, epidemiological and basic research projects (49).
