# Supplementary material for: Food socialization of children with Prader-Willi syndrome: an interdisciplinary problematization
Source: Front Nutr. 2023 Jun 6;10:1177348. doi: 10.3389/fnut.2023.1177348 (PMC10280295; doi:10.3389/fnut.2023.1177348)
Supplement: Supplementary file 1 [file Data_Sheet_1.docx]

Articles used and cited on the problematization

The articles mobilized to construct the Food Social Norms Internalization (FSNI) theory were arranged by topic and date of publication. The table includes the full reference and number for the order of appearance in that article.

| 1. ***Eating disorder in PWS (n33)*** |  |
| --- | --- |
| Prader A, Labhart A, Willi H. Ein Syndrom von Adipositas, Kleinwuchs, Kryptorchidismus und Oligophrenie nach myotoniertigem Zustand im Neugeborenenalter. Schweizerische medizinische Wochenschrift (1956) 6:1260-61. | 26 |
| Nicholls RD, Knoll JH, Butler MG, Karam S, Lalande M. Genetic imprinting suggested by maternal heterodisomy in nondeletion Prader-Willi syndrome. Nature (1989) 342:281-85. | 27 |
| Driscoll DJ, Waters MF, Williams CA, Zori RT, Glenn CC, Avidano KM, et al. A DNA methylation imprint, determined by the sex of the parent, distinguishes the Angelman and Prader-Willi syndromes. Genomics (1992) 13:917-24. | 28 |
| Dimitropoulos A, Feurer ID, Roof E, Stone W, Butler MG, Sutcliffe J, Thompson T. Appetitive behavior, compulsivity, and neurochemistry in Prader-Willi syndrome. Mental Retardation and Developmental Disabilities Research Reviews (2000) 6:125-30. | 2 |
| Whittington JE, Holland AJ, Webb T, Butler J, Clarke D, Boer H. Population prevalence and estimated birth incidence and mortality rate for people with Prader‐Willi syndrome in one UK Health Region. Journal of Medical Genetics (2001) 38:792-98. | 17 |
| Goldberg DL, Garrett CL, Van Riper C, Warzak WJ. Coping with Prader-Willi syndrome. Journal of the American Dietetic Association (2002) 102:537-42. | 46 |
| Dykens EM, Maxwell MA, Pantino E, Kossler R, Roof E. Assessment of hyperphagia in Prader‐Willi syndrome. *Obesity* (2007) 15:1816-26. | 24 |
| Skryabin BV, Gubar LV, Seeger B, Pfeiffer J, Handel S, Robeck T, et al. Deletion of the MBII-85 snoRNA gene cluster in mice results in postnatal growth retardation. PLoS genetics (2007) 3:e235. | 30 |
| Goldstone AP, Holland AJ, Hauffa BP, Hokken-Koelega AC, Tauber M. Speakers at the Second Expert Meeting of the Comprehensive Care of Patients with PWS: Recommendations for the diagnosis and management of Prader-Willi syndrome. The Journal of Clinical Endocrinology & Metabolism (2008) 93:4183-97. | 21 |
| Molinas C, Cazals L, Diene G, Glattard M, Arnaud C, Tauber M; French Reference Centre for PWS (FrRefC-PWS). French database of children and adolescents with Prader-Willi syndrome. BMC Med Genet (2008) 2;9: 89. | 49 |
| Diene G, Mimoun E, Feigerlova E, Caula S, Molinas C, Grandjean H, Tauber M. Endocrine disorders in children with Prader-Willi syndrome - data from 142 children of the French database. Hormone Research in Paediatrics (2010) 74:121-28. | 22 |
| Schaller F, Watrin F, Sturny R, Massacrier A, Szepetowski P, Muscatelli F. A single postnatal injection of oxytocin rescues the lethal feeding behaviour in mouse newborns deficient for the imprinted Magel2 gene. Human Molecular Genetics (2010) 19:4895-4905. | 33 |
| Allen K. Managing Prader-Willi syndrome in families: An embodied exploration, Social Science & Medicine (2011) 72:460-68. | 47 |
| Miller JL, Lynn CH, Driscoll DC, Goldstone AP, Gold JA, Kimonis V. Nutritional Phases in Prader-Willi Syndrome. American Journal of Medical Genetics (2011) 155A:1040-49. | 20 |
| Cassidy S, Schwartz S, Miller J, Driscoll D. Prader-Willi syndrome. *Genetics in Medicine* (2012)14:10-26. | 23 |
| Bieth E, Eddiry S, Gaston V, Lorenzini F, Buffet A, Auriol FC, et al. Highly restricted deletion of the SNORD116 region is implicated in Prader-Willi Syndrome. European Journal of Human Genetics (2014) 23:252-55. | 31 |
| Tauber M, Diene G, Mimoun E, Çabal-Berthoumieu S, Mantoulan C, Molinas C, et al. Prader-Willi syndrome as a model of human hyperphagia. Frontiers of Hormone Research (2014) 42:93-106. | 3 |
| Fehnel SE, Brown TM, Nelson L, Chen A, Kim DD, Roof E, Dykens EM. Development Of The Hyperphagia Questionnaire For Use In Prader-Willi Syndrome Clinical Trials. Value in Health (2015) 18:A25-A25. | 25 |
| Meziane H, Schaller F, Bauer S, Villard C, Matarazzo V, Riet F, et al. An Early Postnatal Oxytocin Treatment Prevents Social and Learning Deficits in Adult Mice Deficient for *Magel*2, a Gene Involved in Prader-Willi Syndrome and Autism. Biological Psychiatry (2015) 78:85-94. | 34 |
| Tauber M, Thuilleaux D, Bieth E. Le syndrome de Prader-Willi en 2015. Médecines/sciences (2015) 10:853-60. | 1 |
| Beauloye V, Diene G, Kuppens R, Zech F, Winandy C, Molinas C, et al. High unacylated ghrelin levels support the concept of anorexia in infants with Prader-Willi syndrome, Orphanet Journal of Rare Diseases (2016) 11:1-8. | 38 |
| Maillard J, Park S, Croizier S, Vanacker C, Cook JH, Prevot V, et al. Loss of Magel2 impairs the development of hypothalamic Anorexigenic circuits. Human Molecular Genetics. (2016) 25:3208-15. | 35 |
| Salles J, Strelnikov K, Carine M, Denise T, Laurier V, Molinas C, et al. Deficits in voice and multisensory processing in patients with Prader-Willi syndrome. Neuropsychologia (2016) 85:137-47. | 87 |
| Bar C, Diene G, Molinas C, Bieth E, Casper C, Tauber M. Early diagnosis and care is achieved but should be improved in infants with Prader‐Willi syndrome. Orphanet Journal of Rare Diseases (2017) 12:1-6. | 18 |
| Burnett LC, LeDuc CA, Sulsona CR, Paull D, Rausch R, Eddiry S, et al. Deficiency in prohormone convertase PC1 impairs prohormone processing in Prader-Willi syndrome. The Journal of Clinical Investigation (2017) 127:293-305. | 37 |
| Polex-Wolf J, Lam BY, Larder R, Tadross J, Rimmington D, Bosch F, et al. Hypothalamic loss of Snord116 recapitulates the hyperphagia of Prader-Willi syndrome. The Journal of Clinical Investigation (2018) 128:960-69. | 32 |
| Ates T, Oncul M, Dilsiz P, Cansu Topcu I, Civan Civas C, Ikbal Alp M, et al. Inactivation of Magel2 suppresses oxytocin neurons through synaptic excitation-inhibition imbalance. Neurobiology of Disease (2019) 121:58-64. | 36 |
| Butler MG, Hartin SN, Hossain WA, Manzardo AM, Kimonis V, Dykens E. et al. Molecular genetic classification in Prader-Willi syndrome: a multisite cohort study. Journal of Medical Genetics (2019) 56:149-53. | 29 |
| Jennings JH, Kim CK, Marshel JH, Raffiee M, Ye L, Quirin S, et al. Interacting neural ensembles in orbitofrontal cortex for social and feeding behaviour. Nature (2019) 565:645-49. | 42 |
| Tauber M, Coupaye M, Diene G, Molinas C, Valette M, Beauloye V. Prader-Willi syndrome: A model for understanding the ghrelin system. Journal of Neuroendocrinology (2019) 31:e12728. | 39 |
| Carter CS, Kenkel WM, MacLean EL, Wilson SR, Perkeybile AM, Yee JR, et al. Is Oxytocin “Nature’s Medicine”? Pharmacological Reviews (2020) 72:829-61. | 40 |
| Salles J, Lacassagne E, Eddiry S, Franchitto N, Salles JP, Tauber M. What can we learn from PWS and SNORD116 genes about the pathophysiology of addictive disorders? Molecular Psychiatry (2020) 26:51-59. | 19 |
| Tauber M, Hoybye C. Endocrine disorders in Prader-Willi syndrome: a model to understand and treat hypothalamic dysfunction. Lancet Diabetes and Endocrinology (2021) 9:235-46. | 41 |
| ***2. Food socialization (n26)*** |  |
| Durkheim E. Rules of Sociological Method, Glencoe, III: Free Press (1938[1895]). | 63 |
| Mauss M. Techniques of the Body. Economy and society (1973[1936]) 2:70-88. | 66 |
| Douglas M. Deciphering a Meal. Daedalus: Myth, Symbol and Culture. Journal of the American Academy of Arts and Sciences (1972) 101:61-81. | 50 |
| Bourdieu P. Distinction: A social critique of the judgement of taste. Cambridge, Harvard: Harvard University Press (1984[1979]). | 67 |
| Chiva M. Comment la personne se construit en mangeant. Communications (1979) 31:107-18. | 62 |
| Murcott A. On the social significance of “cooked dinner” in South Wales. Social Science Information (1982) 21:677-96. | 51 |
| Corbeau JP. L’avaleur n’attend pas le nombre des années, ou l’éducation alimentaire. Les Cahiers de l’iforep (1986) 49:47-65. | 72 |
| Drulhe M. L’incorporation. Sociétés (1987) 15:5-6. | 68 |
| Héran F. La seconde nature de l'habitus. Tradition philosophique et sens commun dans le langage sociologique. Revue française de sociologie (1987) 28:385-416. | 69 |
| Fischler C. Food, Self and Identity. Social Science Information (1988) 27:275-92. | 4 |
| Herpin N. Le repas comme institution, compte rendu d’une enquête exploratoire. Revue française de sociologie (1988) XXIX: 503-21. | 65 |
| Chiva M. Cultural aspects of meals and meal frequency. British Journal of Nutrition (1997) 77: S21-S28. | 52 |
| Sobal J. Sociability and meals: Facilitation, commensality and interaction. In: Meiselman H, Dimensions of the Meal: The science, culture, business, and art of eating. Gaithersburg: Aspen (2000) :119-33. | 53 |
| Poulain JP. Sociology of Food. London: Bloomsbury (2017[2002]). | 5 |
| Sirota R. Éléments pour une sociologie de l'enfance. Rennes : Presses Universitaires de Rennes (2006). | 71 |
| Corbeau JP, Poulain JP. Penser l’alimentation, Entre imaginaire et rationalités. Paris : Privat (2008). | 15 |
| Poulain JP. Sociologie de l’obésité. Paris : Presses Universitaires de France (2009). | 101 |
| Diasio N. Children and food: ambivalent connections between risk, moral technologies, and fun. In: De Beaufort I. Vandamme S, Vande Vathorst S. Whose Weight is it Anyway: Essays on Ethics and Eating. Ghent: Acco Publisher (2010): 55-66. | 74 |
| James A, Trine Kjørholt A, Tingstad V. Children, Food and Identity in Everyday Life. New York: Palgrave Macmillan (2010). | 73 |
| Lalanne M, Tibère L. Enquêter sur l’alimentation des enfants : outils et méthode. In: Poulain JP. Dictionnaire des cultures alimentaires. Paris: Presses Universitaires de France (2012) : 461-70. | 76 |
| Dupuy A, Nicklaus S, Schwartz C, Goirand S, Tibère L. Young Children’s Learning about Hunger and Satiety through the Lens of the Norms of Those Who Feed Them. Social Science (2021), 10(8), 292 [Online]: https://doi.org/10.3390/socsci10080292 | 75 |
| Warde A. The practice of eating. Cambridge: Polity (2016). | 16 |
| Fournier T, Poulain JP. La génomique nutritionnelle : (re)penser les liens alimentation-santé à l’articulation des sciences sociales, biomédicales et de la vie. Natures Sciences Sociétés (2017) 25:111-21. | 59 |
| Rochedy A. Note de recherche - Autismes et socialisations alimentaires. Particularités alimentaires des enfants avec un Trouble du Spectre de l’Autisme et ajustements parentaux pour y faire face. ALTER - European Journal of Disability Research (2018) 12:41-49. | 12 |
| Poulain JP. Towards a sociological theory of eating: a review of Alan Warde's “The Practice of Eating”. Anthropology of food (2020) [Online]: http://journals.openedition.org/aof/10866. | 14 |
| Corbeau J.P Reflections for a Sociological Representation of the Eater. Social Sciences (2021) 10(9): 339 [Online]: https://doi.org/10.3390/socsci10090339. | 77 |
| ***3 Neophobia (n15)*** |  |
| Spitz RA. No and yes: on the genesis of human communication. New York: International Universities Press (1957). | 90 |
| Rozin P. The selection of food by rats, humans and other animals. In: Slater PJB, Beer C. Advances in the study Behavior. New York: Academic Press (1976) : 21-76. | 70 |
| Birch LL. Effects of Peer Model’s Food Choices and Eating Behaviors on Preschooler’s Food Preferences. Child Development (1980) 51:489-96. | 6 |
| Fischler C, Chiva M. Food likes, dislikes and some of their correlates in a sample of French children and young adults. In: Booth DA, Conner MT, Leigh Gibson E. Measurement and determinants of food habits and food preferences. Wageningen: Department of Human Nutrition, Agricultural University (1986) p.137-56. | 7 |
| Hanse L. La néophobie alimentaire chez l’enfant. Thèse de doctorat : psychologie. Paris : Paris 10 (1994). | 8 |
| Lecanuet JP, Schaal B. Fetal sensory competences, European Journal of Obstetrics, Gynaecology, and Reproductive Biology (1996) 68:1-23. | 60 |
| Rigal N. La naissance du goût : comment donner aux enfants le plaisir de manger. Paris : Noêsis (2000). | 89 |
| Poulain JP. Néophobie alimentaire. In : Poulain JP. Dictionnaire des cultures alimentaires. Paris : PUF (2012) p.981-83. | 9 |
| Rigal N, Chabanet C, Issanchou S, Monnery-Patris S. Links between maternal feeding practices and children’s eating difficulties. Validation of French tools. Appetite (2012) 58:629-37 | 10 |
| Rochedy A, Poulain JP. Approche sociologique des néophobies alimentaires chez l’enfant. Dialogue (2015) 209:55-68. | 11 |
| Rochedy A, Raynaud JP, Maffre T, Poulain JP. (Dé)formations du processus de néophobie. Une approche sociologique des particularités alimentaires des enfants avec un Trouble du Spectre Autistique. Neuropsychiatrie de l’Enfant et de l’Adolescent (2020) 68:347-55. | 13 |
| Gray DE. Gender and coping: the parents of children with high functioning autism. Social Science & Medicine (2003) 56:631-42. | 43 |
| Dubois L, Farmer A, Girard M, Porcherie M. Family food insufficiency is related to overweight among preschoolers. Social Science & Medicine (2006) 63:1503-16. | 48 |
| Tsaï W, Tsaï J, Shyu YL. Integrating the nurturer-trainer roles: parent and behaviour/symptom management processes for mothers of children with autism. Social Science & Medicine (2008) 67: 1798-806. | 45 |
| Berrocoso S, Amayra I, Lázaro E, Martínez O, López-Paz JF, García M, et al. Coping with Wolf-Hirschhorn syndrome: quality of life and psychosocial features of family carers. Orphanet Journal of Rare Diseases (2020) 15:1-14. | 44 |
| ***4 Psycho-development (n10)*** |  |
| Freud S. Three essays on the theory of sexuality: The 1905 edition. Verso Books (2017[1905]). | 79 |
| Freud S. The infantile genital organization of the libido: A supplement to the theory of sexuality. International Journal of Psycho-Analysis (1924) 5:125-129. | 80 |
| Klein M. The psychoanalysis of children. New York: Grove Press (1960[1932]). | 88 |
| Wallon H. L’évolution psychologique de l’enfant. Paris: Colin (1968[1941]). | 83 |
| Osterrieth P, Piaget J, De Saussure R, Tanner JM, Wallon H, Zazzo R, et al. Le problème des stades en psychologie de l’enfant. Paris: Presses Universitaires de France (1956). | 78 |
| Piaget J. Six psychological studies. New York: Random House (1967[1964]). | 82 |
| Erikson EH. Autobiographic notes on the identity crisis. Daedalus (1970) 99:730-59. | 85 |
| Gratiot-Alphandéry H, Zazzo R. Traité de psychologie de l’enfant. Tome I Histoire et généralités. Paris: Presses Universitaires de France (1970). | 84 |
| Malewska-Peyre H, Tap P. Les enjeux de la socialisation. In: Malewska-Peyre H, Tap P. La socialisation de l'enfance à l'adolescence. Paris: Presses Universitaires de France (1991): 7-17. | 91 |
| Dolto F. Les étapes majeures de l'enfance. Paris: Gallimard (1994). | 81 |
| ***5 Familialisation (n7)*** |  |
| Leitner S. Varieties of familialism: The caring function of the family in comparative perspective. European societies (2003) 5(4): 353-75. | 92 |
| Keppens D. Comment le médical devient familial: obésité et diabète de l'enfant. Doctoral dissertation, Université de Paris 5 (2010). | 98 |
| Martin C. « Être un bon parent ». Une injonction contemporaine. Rennes : Presses de l’École des hautes études en santé publique (2014). | 97 |
| Saraceno C. Varieties of familialism: Comparing four southern European and East Asian welfare regimes. Journal of European Social Policy, 26(4), 314-326. (2016). | 94 |
| Szelewa D. From implicit to explicit familialism: Post-1989 family policy reforms in Poland. In: Auth D, Hergenhan J. Gender and family in European economic policy: Developments in the new millennium. Springer (2016): 129-51. | 95 |
| Eggers T, Grages C, Pfau-Effinger B, Och R. Re-conceptualising the relationship between de-familialisation and familialisation and the implications for gender equality - the case of long-term care policies for older people. Ageing & Society (2020) 40(4): 869-95. | 96 |
| Furmańska-Maruszak A, Suwada K. Familialisation of Care in European Societies: Between Family and the State. In: Castrén AM, Česnuitytė V, Crespi I and al. The Palgrave Handbook of Family Sociology in Europe. Cham: Springer International Publishing (2021): 205-21. | 93 |
